# Borders, cross-border cooperation and depopulation: the case of the Spanish-Portuguese North-Central Border

**DOI:** 10.12688/openreseurope.16319.1

**Published:** 2023-11-20

**Authors:** PABLO PODADERA-RIVERA, FRANCISCO J. CALDERON-VAZQUEZ

**Affiliations:** 1 Department of Applied Economics (Public Finance, Economic Policy and Political Economy), University of Malaga, Málaga, Andalusia, 29071, Spain

**Keywords:** institutional and administrative coordination, Borders, Cross-Border Cooperation, Depopulation, European Union

## Abstract

**Background:**

Cross-border cooperation has been (and still is) one of the main assets of the acquis communitiare in everything that refers to cross-border development. In fact, development initiatives aimed at addressing the problems of intra-European border areas could have better results if they were concerted (horizontally coordinated) and executed jointly by the governments, national, and local actors from both border sides, to improve their populations’ quality of life. The study goal is to provide new contributions that enrich critical reflection and debate on the issue of depopulation, in order to propose economic policy recommendations within a framework of cross-border institutional cooperation and inter- and intra-governmental coordination of political actors involved.

**Methods:**

We prepared a descriptive analysis of its evolution during the period 1960-2020, with three essential variables: a) population evolution border based on the losses or gains verified with respect to 1960; b) critical population mass existing in 2020 based on the indicator of population density (inhabitants per square kilometer), and c) level of population aging based on the indicator percentage of population over 65 years of age over total population. Our descriptive analysis exclusively covers the border municipalities located along the both sides of the Spanish-Portuguese north-central border line, a total of 49 border municipalities (nine Portuguese and 40 spanish).

**Results:**

The results show the existence of this recessive demographic continuum, which extends to both sides of the border, causes the need to face the depopulating phenomenon from a transnational and European perspective, in the sense that ‘national’ solutions to the phenomenon will not be able to offer answers common and consistent to a phenomenon as complex as depopulation.

**Conclusions:**

It is necessary to reinforce the existing cross-border cooperation, providing it with greater operability at the level of institutional and administrative coordination, deepening the multilevel governance of the territory.

## Plain language summary

Among these imbalances that affect border areas, both depopulation and population aging appear as very current phenomena that affect many continental territories of the European Union and, in particular, border areas such as the Spanish-Portuguese Central-North border. Our work explores the relationships between the phenomenon of depopulation and the cross-border cooperation actions are carried out on the Spanish-Portuguese Central-North border.

Our work proposes a descriptive-evolutionary analysis of the situation of the border population in the period 1960–2020, taking as a territorial basis the border municipalities of the Spanish-Portuguese central-north border, covering a total of 49 border municipalities (nine Portuguese and 40 Spanish). The analysis considers three essential variables: population evolution depending on the border on the losses or gains verified with respect to 1960; critical mass of existing population in 2020 based on the indicator of population density (inhabitants per square kilometer), and level of population aging based on the indicator percentage of population over 65 years of the total population.

The final purpose of this work is to provide new materials that enrich reflection and critical debate on the issue of depopulation, in order to propose Economic Policy recommendations, within a framework of cross-border institutional cooperation and inter and intragovernmental coordination.

In this sense, the results show that the existence of a recessive demographic continuum, which extends to both sides of the Spanish-Portuguese central-north border, which causes the need to face the depopulation phenomenon from a transnational and, above all, European perspective. Since the ‘national’ solutions to the depopulating phenomenon will not be able to offer common and coherent answers to a problem as complex as depopulation. For this, it is necessary to reinforce the existing cross-border cooperation, making it more operational at the level of institutional and administrative coordination, deepening the multilevel governance of the territory.

## Introduction

The Spanish-Portuguese border, the Iberian ‘Raya’ (stripe) and, within it, the Central-Northern Stripe have encompassed throughout their profuse history each and every one of the border typologies, embodying both types of borders, whether ‘opaque’ or ‘open’, evolving through the centuries from a warlike and conflictual medieval past to the internal community border that it is today.

It is a secular border, since its essential demarcation is made with the Treaties of Zamora (1143), Badajoz (1267) and Alcañices (1297), if we take the latter as a reference, the Central-North Stripe has already exceeded the 725 years of existence, resulting in the oldest border in Europe.

Within the motley territorial set of the Iberian Stripe, the Central-North section of the Stripe runs along a 320km shared border, covering the Luso-Hispanic borderlands between the Autonomous Community of Castilla y Leon, the Central Region and the Portuguese North Region. At the NUTS III administrative level, the Reya Centro-Norte covers the Spanish Provinces of Salamanca and Zamora and the Portuguese Districts of Guarda ^1^ and Braganza ^2^. At the Municipal level, from the Portuguese side, the Central-North Line runs from the Sabugal council (southern limit) to Vinhais (northern limit) and, from the Spanish side, from Navasfrias (SA) to Hermisende on the northern limit.

A common place in studies on the Central-North Raya is the problem of depopulation ( Burillo, 2020; Cabero Diéguez, 1993; Cabero Diéguez, 1996; Hortelano & Mansvelt, 2017; Moreira & Rodrigues, 2000; Pascoal, 2000; Senabre, 2013), normally underlining the existence of important socio-demographic restrictions, restrictions that reach the category of "structural" ( Bustos Gisbert, 2005). Given that these restrictions represent a substantive limit in the processes of territorial development (especially in the coordinates of Endogenous Development) ^3^, it seems opportune and significant to compare the current demographic situation of the Central-North Raya, three decades after the signing of the first agreements. of cross-border cooperation (1992), considering to what extent cross-border practices have influenced border population dynamics.

For their part, the integration processes, in general, and that of the European Union (EU), in particular, require large doses of (territorial) unity in favor of a growing economic, social and territorial cohesion, which entails major governance challenges multilevel ( Podadera & Calderón, 2018), both within the different Member States and across national borders, particularly the Spanish-Portuguese North-Central border.

### Study objectives

Therefore, the ultimate goal of this work is to provide new contributions that enrich critical reflection and debate on the issue of depopulation, in order to propose Economic Policy recommendations, within a framework of cross-border institutional cooperation and inter- and intra-governmental coordination. of political actors involved.

Likewise, as specific objectives, it is intended, on the one hand, to contrast the situation of the population in everything that refers to population critical mass (evolution, volume, density) and levels of aging (population over 65 years) in the Spanish-Portuguese North-Central border, and, on the other hand, verify the economic-demographic relations.

## Methods

Our work could be framed in the category of research article (although it incorporates elements of review, reflection and recommendations), by proposing a descriptive analysis of the population situation in the municipalities of Spanish-Portuguese North-Central border, an analysis prepared from the collection of data from official statistical sources ^4^, relating the depopulation process with cross-border cooperation activities, contrasting the elements of institutional collaboration observed in the empirical evidence.

In order to contrast the situation of border population on both sides of Spanish-Portuguese North-Central border, we have prepared a descriptive analysis of its evolution during the period 1960–2020, taking into account three essential variables: a) population evolution border based on the losses or gains verified with respect to 1960; b) critical population mass existing in 2020 based on the indicator of population density (inhabitants per square kilometer) and c) level of population aging based on the indicator percentage of population over 65 years of age over total population. Our descriptive analysis covers only (and exclusively) the border municipalities located along the both sides of the Spanish-Portuguese north-central border line, a total of 49 border municipalities (nine Portuguese and 40 Spanish).

In the Spanish case, the demographic data used comes from the National Institute of Statistics (INE) specifically from each source as detailed below:

1) Official population figures from Spanish municipalities: Revision of the Municipal Register. Results: https://ine.es/dynt3/inebase/es/index.htm?padre=525; Specifically from border municipalities of Salamanca Province uni https://ine.es/jaxiT3/Tabla.htm?t=2891&L=0 and the ones from Zamora province https://ine.es/jaxiT3/Tabla.htm?t=2906&L=0.

Given the complexity of the analysis to be carried out, we have complemented the official data with the database of institutes specialized in territorial statistics. In the specific case of the aging data of the Spanish border municipalities, we have supplemented the official data with those from the Matrix Foundation and its interactive territorial maps ( https://fundacionmatrix.es/envejecimiento-poblacional-a-escala-municipal-de-spain-geographical-trends-and-associated-factors/)

In the Portuguese case, the demographic data used in the preparation of Table 1 and Table 2 come from the Statistics Portugal database of the Portuguese National Institute of Statistics (INE.pt). Specifically, for the Portuguese border municipalities the source of the original data is Population Census:

**Table 1.** Administrative-territorial scope of the study.

SPANISH-PORTUGUESE NORTH-CENTRAL BORDER					
Portuguese NUTS 2	NUTS 3 Number code	Portuguese NUTS 3	Spanish NUTS 3	NUTS 3 Number Code	Spanish NUTS 2
**PT16 CENTRO**	**PT16J**	**Beiras e Serra ** **da Estrela**	**Salamanca Province**	**ES415**	**CASTILLA-LEON ES41**
**PT11 NORTE**	**PT11D**	**Douro**	**Salamanca Province**	**ES415**	**CASTILLA-LEON ES41**
**PT11 NORTE**	**PT11E**	**Terras de Trás-** **os-Montes**	**Zamora Province**	**ES419**	**CASTILLA-LEON ES41**

Source: authors based on Eurostat data ^6^

**Table 2.** CENTRAL-NORTHERN BORDER SECTION A: Portuguese districts of Guarda and Braganza / Province of Salamanca. (Autonomous Community of Castilla-Leon).

CENTRAL-NORTHERN SPANISH PORTUGESE BORDER							
DISTRICT OF GUARDA (PORTUGAL)				PROVINCE OF SALAMANCA (SPAIN)			
PORTUGUESE MUNICIPALITIES				SPANISH MUNICIPALITIES			
Municipality	Population Density ^7^ (H/Km²)	Evolution Population Amount (%) (1960 -2020)	Aging Level (%) > 65 (2019	Population Density (H/ Km²)	Evolution Population Amount (%) (1960 -2020	Aging Level (%) > 65 (2019	Municipality
**Sabugal**	**12,8**	**-72%**	**37,13%**	**7,04**	**-77%**	**42.4**	**Navasfrias (SA)**
				**4,32**	**-78%**	**50.3**	**Casillas de Flores**
				**3.53**	**-85%**	**44.9**	** Alberguería Argañán**
				**6,10**	**-83%**	**45.7**	**Alamedilla**
**Almeida**	**11,2**	**-64%**	**36,14%**				
				**2,29**	**-76%**	**40.0**	**Espeja**
				**21,12**	**-12%**	**23.0**	**Fuentes de Oñoro**
				**2,11**	**-90%**	**57.0**	**Alameda de Gardón**
				**6,70**	**-65%**	**44.5**	**Aldea del Obispo**
				**4,77**	**-78%**	**47.2**	**Villar de Ciervo**
**Figueira Castel ** **Rodrigo**	**11**	**-58%**	**31,02%**	**3,57**	**-73%**	**30.8**	**Bouza (La)**
				**2,11**	**-88%**	**53.2**	**Puerto Seguro**
				**4,51**	**-81%**	**44.4**	**Ahigal de Aceiteros**
				**4,37**	**-81%**	**45.3**	**Sobradillo**
				**8,04**	**-75%**	**48.2**	**La Fregeneda**
**BRAGANZA DISTRICT**							
**Freixo de** ** Espada a Cinta**	**13.5**	**-55%**	**30,44%**				
				**7,43**	**-66%**	**39.8**	**Hinojosa de Duero**
				**6,41**	**-83%**	**42.0**	**Saucelle**
				**9,37**	**-69%**	**46.6**	**Vilvestre**
				**6,38**	**-83%**	**60.8**	**Mieza**
				**27,24**	**-64%**	**39.5**	**Aldeadávila**
**Mogadouro**	**11,1**	**-57%**	** 35,5%**				
				**14.42**	**-74%**	**50.0**	**Masueco**
				**9,20**	**-71%**	**43.5**	**Pereña de la Ribera**
				**8,21**	**-47%**	**45.2**	**Villarino de los Aires**
				**18,2**	**-70%**	**43.4**	**Fermoselle (ZA)**

Source: Authors based on data from INE.es and INE.pt (2022)

1) https://censos.ine.pt/xportal/xmain?xpgid=censos21_dados_finais&xpid=CENSOS21&xlang=pt

2) https://censos.ine.pt/xportal/xmain?xpgid=censos21_populacao&xpid=CENSOS21

As in the Spanish case, we have also complemented the official data related to population aging with data from territorially specialized statistical institutes and publications ( Curbelo, 2020). The institutions involved are INE.pt and ISEG - Lisbon School of Economics & Management

Based on these sources, we have configured the generic population matrices for the period 1960–2020, including all the border municipalities (see Table 2 and Table 3) of the Central-North Raya. Given the large number of municipalities to be covered in our study, the individualized collection of data by municipality on both sides of the north-central border has required a great effort to collect data, not always as homogeneous as we would have liked, but which have allowed us to create this mosaic map encompasses a lot of information that could be improved and developed by further studies.

**Table 3.** CENTRAL-NORTHERN BORDER SECTION B: : Portuguese district of Braganza / Province of Zamora (Autonomous Community of Castilla-Leon).

DISTRICT OF BRAGANZA (PORTUGAL)				PROVINCE OF ZAMORA (SPAIN)			
PORTUGUESE MUNICIPALITIES				SPANISH MUNICIPALITIES			
Municipality	Population Density (H/Km²)	Evolution Population Amount (%) (1960–2020)	Aging Level (%) > 65 (2019	Population Density ^8^ (H/Km²)	Evolution Population Amount (%) (1960–2020	Aging Level (%) > 65 (2019	Municipality
**Miranda** **do Douro**	**14,0**	**-64%**	**33,47%**	**18,2**	**-70%**	**43.4**	**Fermoselle (ZA)**
				**4,5**	**-35%**	**49.0**	**Villar del Buey**
				**5,85**	**-49%**	**49.3**	**Fariza**
				**7,72**	**-64%**	**39.2**	**Torregamones**
				**4,64**	**-77%**	**49.3**	**Villardiegua la ** **Ribera**
				**5,97**	**-57%**	**42.6**	**Fonfría**
				**19,65**	**-31%**	**33.7**	**Alcañices**
**Vimioso**	**8.4**	**-69%**	**34,63%**				
				**6,95**	**-77%**	**47.6**	**Rábano de Aliste**
				**9,77**	**-57%**	**40.4**	**Trabazos**
**Braganza**	**28,6**	**-11%**	**25,19%**				
				**2,33**	**-79%**	**59.4**	**Figueruela de ** **Arriba**
				**2,81**	**-76%**	**49.7**	**Manzanal de Arriba**
				**17,59**	**+5%**	**25.0**	**Puebla de Sanabria**
				**2,48**	**-81%**	**47.5**	**Pedralba la ** **Pradería**
				**3,01**	**-73%**	**38.1**	**Requejo**
				**2,33**	**-81%**	**49.4**	**Hermisende**
**Vinhais**	**11.5**	**-71%**	**42,19%**				

Source: Authors based on data from INE.es and INE.pt (2022)

It is never easy to compare Spain and Portugal, even though there are so many similarities (EU members, shared past, joint future, etc.) given that both their administrative structures and their territorial organization are substantially different: While Spain today is a country Strongly decentralized, Portugal maintains high levels of centralization, with the ‘Regiões’ having an essentially statistical functionality (for EU statistical purposes). The axis of the Portuguese political-administrative organization is the municipality or concelho, an entity that is territorially and administratively subdivided into parishes. This term could be translated, depending on the case, as 'civil parish', 'district', 'district', or 'neighborhood'. The parish is governed by the parish council, equivalent to the Spanish town hall. As the parish is the administrative subdivision of the Portuguese municipality, it will necessarily have to be considered in any territorial analysis of Portugal. Parishes and municipalities are grouped into ‘districts’.

In the Spanish case, the municipality constitutes the basic entity of the Spanish territorial organization. Municipalities can be associated in larger territorial entities with legal personality such as counties, associations, etc. There are also smaller local entities that, even presenting some similarities, would not be, in any case, equivalent to the Portuguese parishes. The municipalities are grouped into provinces (50 throughout the national territory). Each autonomous community (17 in total) is made up of one or several provinces. The set of autonomous communities (Comunidades Autonomas - CCAA) constitutes that peculiar regional unitary State or ‘autonomous state’ that we call Spain.

In order to carry out the Study effectively, we have taken advantage of the synergies derived from the Nomenclature of Territorial Units for Statistics (NUTS) ^5^ classification established by the European Union for community territorial harmonization. Given that the Portuguese NUTSIII ^9^ would be equivalent to the Spanish provinces, the territorial map of the Study would be the following:

### Analyzing economic-demographic relationships

For greater clarity and precision of the study, we have segmented the set of border territories of the Central-North Strip into two large sections. Section A ( Table 2) that covers an extension of 170 km of shared border, where the border line winds along along the Portuguese-Hispanic border, separating, at the NUTS II level, the Community Autonomous Region of Castilla y Leon (ES41) from the neighboring Portuguese Central (PT16) and North (PT11) Regions, while at NUTS Level III the border separates the lands of the Spanish Province of Salamanca from the Portuguese Subregions of Beiras and Serra de Estrela (PT16J), and Douro (PT11D). Therefore, Section A includes all the municipalities (and their Freguesías) of the Portuguese Districts of Faro and Beja, with the bordering municipalities of the Province of Huelva.

On the other side, Section B ( Table 2) of the Raya Centro-Norte crosses longitudinally, along a 150 km shared border, the lands of the Autonomous Community of Castilla y Leon (ES41), bordering the North Region (PT11) Portuguese. While at NUTS III level, Section B includes the Spanish Province of Zamora (ES419), bordering the Portuguese Sub-region of Terras de Tras os Montes (PT11E). Therefore, Section B covers the Portuguese municipalities (and their parishes) of district of Braganza with the bordering municipalities of Zamora.

For an easy referenced reading of the data, we have arranged the information within the Tables in double entry format, so that each Portuguese municipality appears in a parallel line with its bordering Spanish municipalities, with which it shares the same border. The descriptive analysis follows a South-North orientation, from the municipality of Sabugal in Portugal, bordering the Hispanic municipality of Navasfrias, to the Portuguese municipality of Vinhais, bordering the municipality of Hermisende, already on the border with the Autonomous Community of Galicia. in Spain.

## Results

### The Spanish side of Spanish-Portuguese North-Central border

**Figure 1.**
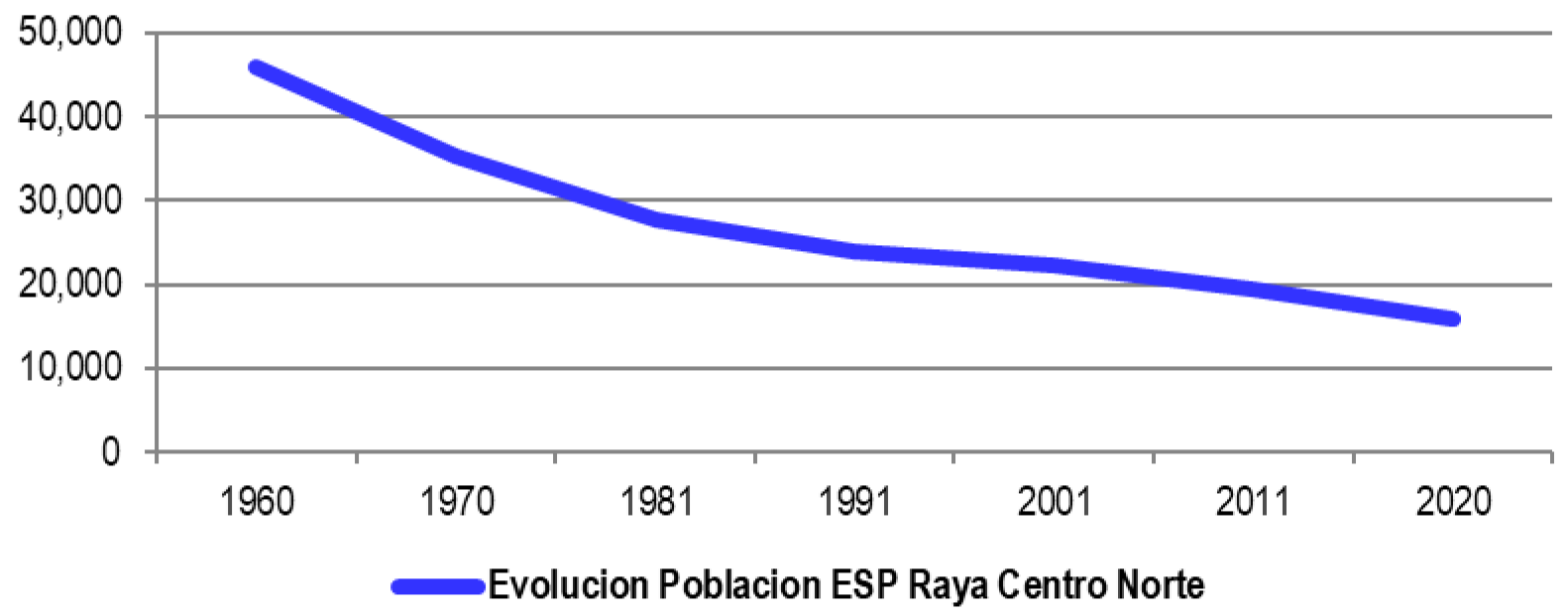
Evolution of the population in Spanish municipalities of the Central-North Raya (1960–202).

**Figure 2.**
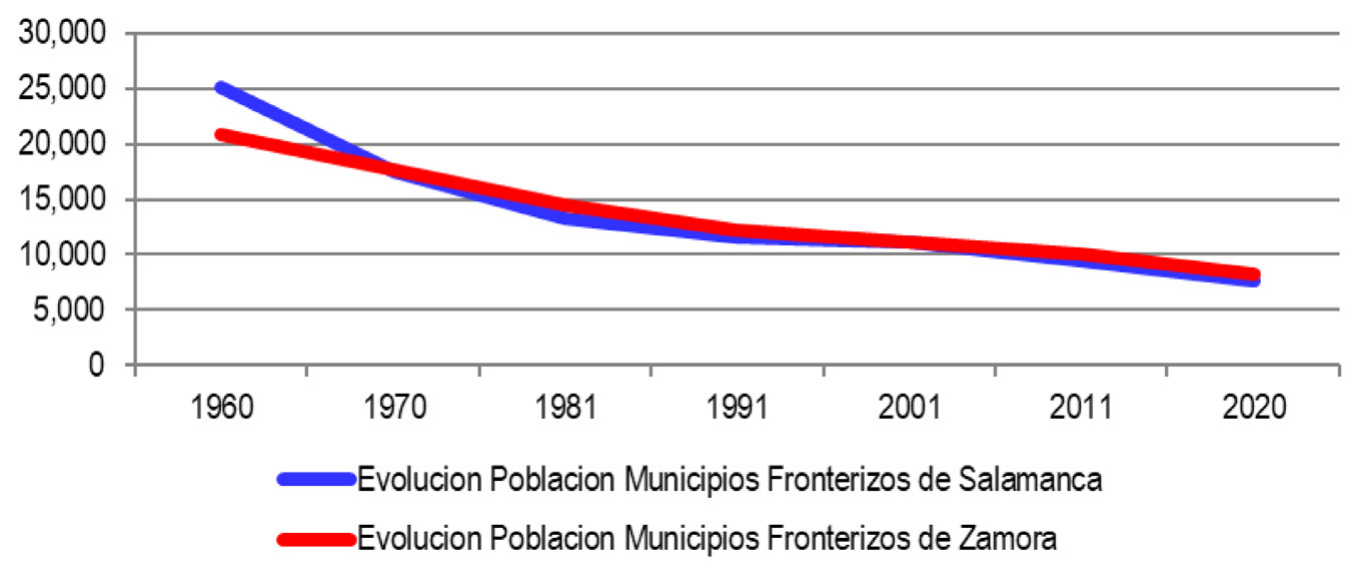
Evolution of the population in municipalities of Salamanca (1960–2020).

**Figure 3.**
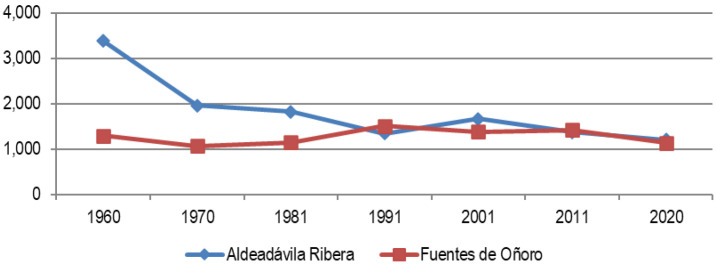
Evolution of the population in municipalities of Salamanca resilient to depopulation (1960–2020).

The following graphics do not seem to leave room for doubt; The collapse of the population in the Spanish border municipalities in the period 1960–2020 is very evident ( Figure 1 and Figure 2), going from housing a population of almost 46,000 inhabitants to move around 16,000. For this reason, the Spanish border municipalities of the Central-North Raya face a situation of accelerated depopulation as we have verified in the descriptive analysis ( Table 2 and Table 3). Within this generic people desertification panorama, we find few (or very few) exceptions; only the municipalities of Aldeadávila and Fuentes (due to their economic dynamics and their structural profiles, as we have contrasted in the analysis) show a certain resilience to the depopulating impact ( Figure 3) with gains or stabilization in the period 1980–2011, although in the last decade its decline shows a worsening of the situation.

**Figure 4.**
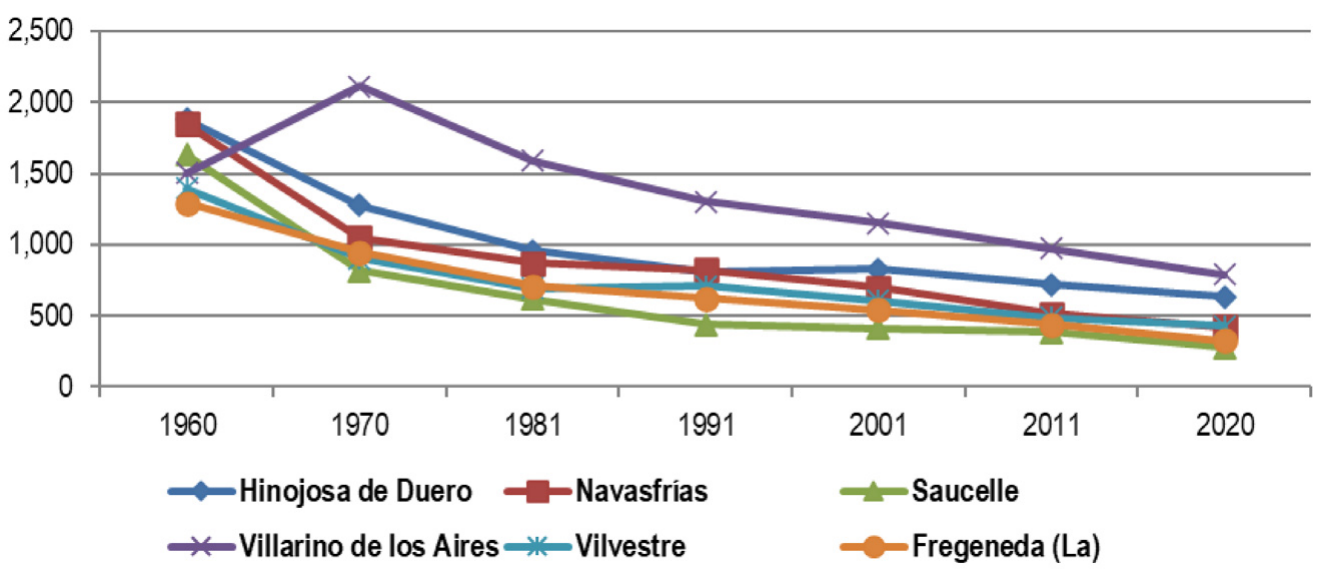
Evolution of the population in municipalities of Salamanca affected by depopulation (1960–2020).

On the contrary, in the remaining municipalities the impact of depopulation is evident as traumatic. Thus, in the Figure 4 includes several border municipalities that in 1960 comfortably exceeded 1,300 inhabitants and that in 2020 move, almost all, below 500 inhabitants.

**Figure 5.**
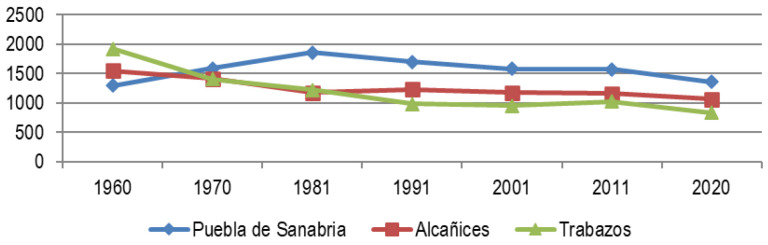
Evolution of the population in Zamora municipalities most resilient to depopulation (1960–2020). Source: Authors based on data from INE.es (2022).

**Figure 6.**
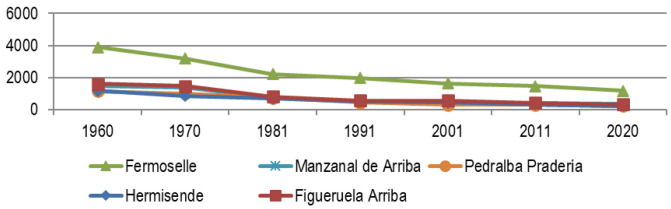
Evolution of the population in municipalities of Zamora affected by depopulation (1960–2020). Source: Authors based on data from INE.es (2022).

In the case of Raya Zamorana ( Figure 5), the situation is similar, although we found three municipalities resilient to depopulation, as we contrasted in point 2.2, Puebla de Sanabria, Alcañices and Trabazos. Its resilience is based on its endowment of services, equipment, infrastructures and economies that make possible a certain quality of life that does not exist in other border towns, although in the last decade even the resilient ones have diminished. Figure 6 shows us the evolution of non-resilient municipalities, 5 municipalities with more than 1,000 inhabitants in 1960, which in 2020 are below 500 inhabitants, a less abrupt implosion than in the Salamanca case, but equally lethal. In addition, a certain stabilization is observed between 1991–2011, after which they slide down the slope again, leaving their population reduced to a few hundred and their uncertain future filling with shadows.

### The Portuguese side of Spanish-Portuguese North-Central border

**Figure 7.**
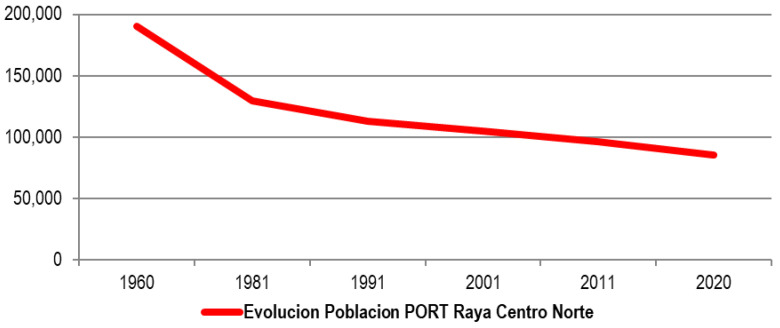
Evolution of the total population in Portuguese municipalities of the Central-North Line (1960–2020).

In line with the maximum, the Portuguese part of the Central-North Raya withstands depopulation pressure better than the Spanish one, although as we have verified in the descriptive analysis, the contractive trend becomes evident, as evidenced by the Figure 7, going from more than 190,000 inhabitants in 1960 to 85,000 in 2020. With the exception of Braganza, practically in all the municipalities, a reiterative pattern of more or less abrupt losses (1960–1981) as in the cases of sabugal and Vinhais, followed by a smooth and gradual decline.

**Figure 8.**
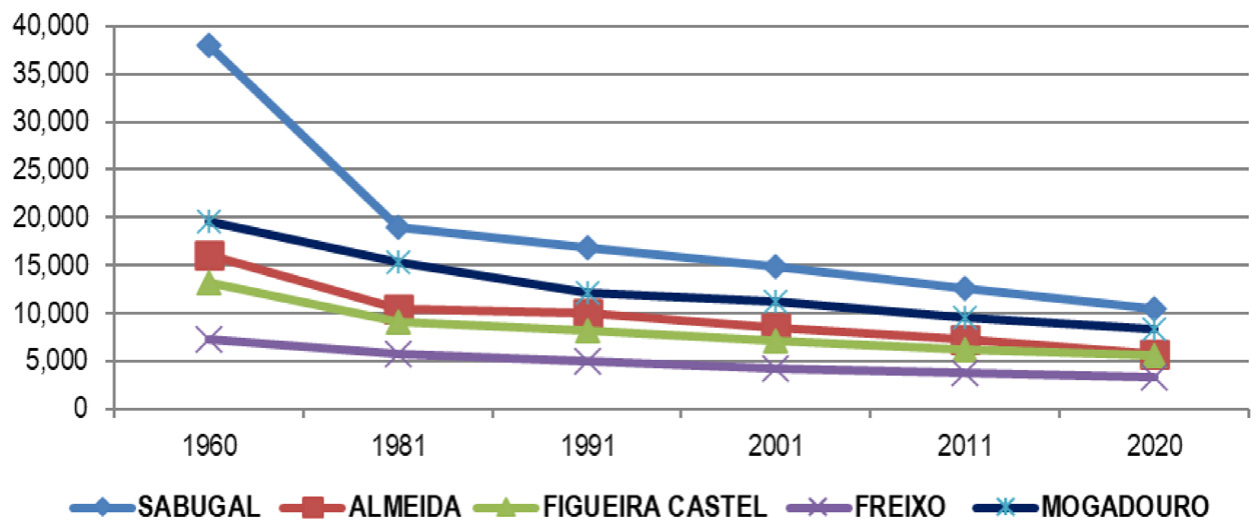
Population evolution of Portuguese municipalities section A Raya Center-North (1960–2020). Source: Authors based on data from INE.pt (2022).

**Figure 9.**
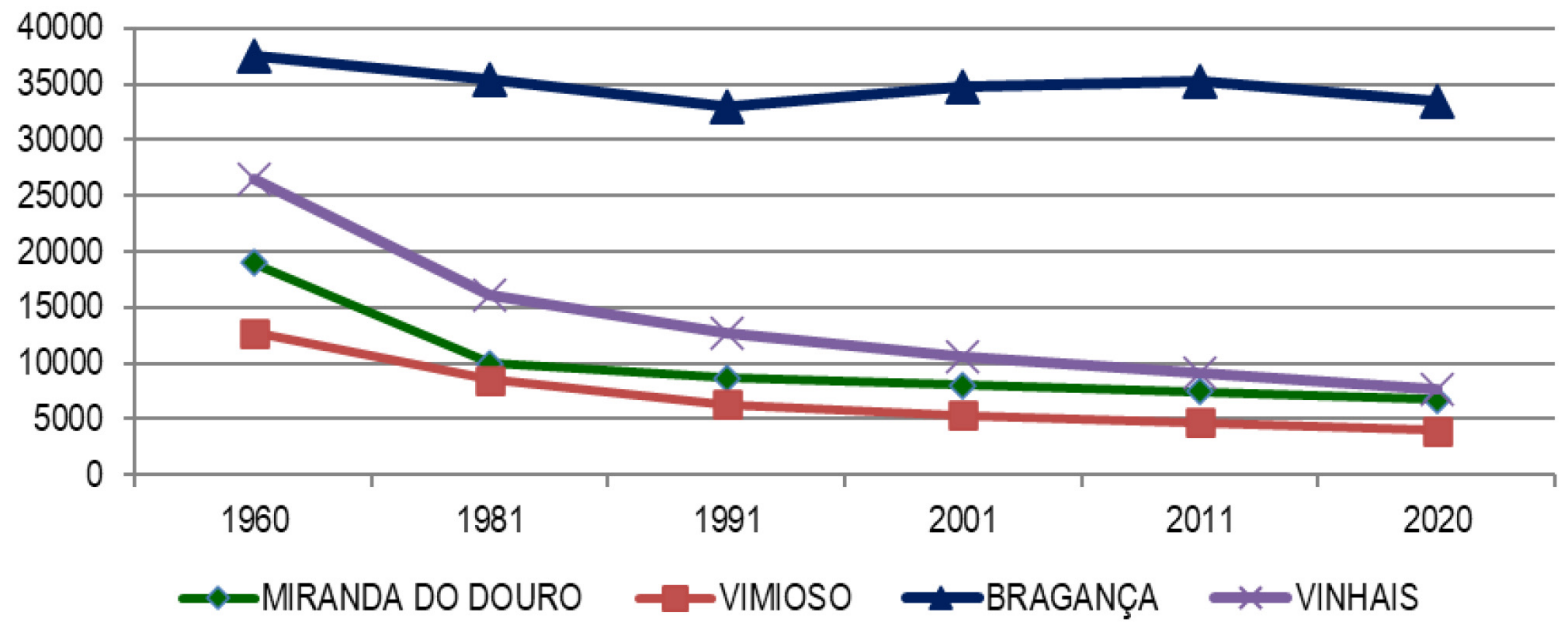
Population evolution of Portuguese municipalities section B Central-North line (1960–2020). Source: Authors based on data from INE.pt (2022).

Said decline is due, as evidenced by Table N. 4 (for the year 2020) to the constant erosion of population volume (via negative vegetative population growth), in all municipalities without exception. For this reason, although the Portuguese side resists the attacks of depopulation better than the Spanish one, it does not mean that it is exempt from said problems, depopulation being both a cause and an effect on the Portuguese side ( Figure 8 and Figure 9).

**Table 4.** Natural balance and migratory balance in Portuguese municipalities Raya Centro-Norte (1960–2020).

Municipality	born	Deceased	natural balance	Migratory balance	Totals
**SABUGAL**	48	308	**-260**	**+200**	**-60**
**ALMEIDA**	18	152	**-134**	**+90**	**-44**
**FIGUEIRA CASTEL**	36	105	**-69**	**+72**	**+3**
**FREIXO**	18	93	**-75**	**+16**	**-59**
**MOGADOURO**	36	154	**-118**	**+12**	**-106**
**MIRANDA DO DOURO**	30	124	**-94**	**+27**	**-67**
**VIMIOSO**	22	114	**-92**	**+27**	**-65**
**BRAGANÇA**	251	585	**-334**	**+168**	**-166**
**VINHAIS**	32	179	**-147**	**+10**	**-137**

Source: Authors. Data from https://ugeo.urbistat.com/AdminStat/en/pt/demografia/popolazione (2022) Note: From the difference of the data of born and deceased (provided), the natural balance is obtained and from the difference of this with the migratory balance (provided) the totals are obtained.

The situation of population concentration in the municipal headquarters of the Portuguese Concelhos, which has been repeated in our analysis along the entire border line, indicates to us to what extent the current depopulation of the rural border territories (at least in the Portuguese side) seems to have a lot to do with the reassignment of the population from the most peripheral and lagging rural areas to urban centers (or with urban functions) endowed with public and private services, equipment and facilities (educational, health, administrative, financial, banks, pharmaceuticals, repair shops, shops, etc.) as well as economic activities other than agriculture. In short, the necessary cocktail for a ‘better’ life that, on the one hand, ‘retains’ the current residents, preventing them from abandoning the territory and, on the other, ‘attracts’ the older population to these ‘urban functionality’ areas. young and ready.

This bet of the Portuguese State for the border urban or pseudo-urban areas used to either ‘anchor’ the population, or a significant part to the border territory, seems clear in the case of Braganza and the respective municipal headquarters. Certainly, said strategy omits rural areas and border parishes for which only cost-reducing administrative instruments (such as Unions of Parishes) seem to be considered. On the Spanish side, it does not seem that there is anything similar or close to a state strategy comparable to the Portuguese one (even with all its shadows). What we have verified in the descriptive analysis is a wide casuistry where municipalities, associations, counties and councils try to carry out territorial, cross-border or local development initiatives normally oriented towards territorial tourism development.

In fact, the turn towards tourist activities (with greater intensity and results on the Spanish side), quite contrasting in our analysis, seems to be consolidating along the entire border line, which has in its historical past and in its rich ecological context -natural great assets for the future. The accumulation of important natural, environmental, historical-artistic, eno-gastronomic, cultural and anthropological resources along the entire Central-North Stripe make it a tourist destination with high potential.

In this sense, the accumulation of environmental resources (Douro International Natural Park, Arribes do Duero Natural Park, Special Protection Areas for Birds (ZEPA), Special Conservation Areas (ZEC) of the Natura 2000 Network; Reserve of the Meseta Ibérica Cross-border Biosphere (Unesco; Malcata Natural Reserve, ‘El Rebollar’ Natural Area among others) is simply extraordinary, a natural, environmental and landscape treasure of great economic significance that enables the implementation of various tourist varieties (Rural Tourism, Environmental , Fluvial with nautical uses, fluvial beaches, etc.). Resources that, like powerful magnetic fields, can attract thousands of visitors, configuring the subsequent tourist demand whose needs have to be met by the offer (accommodation, gastronomy, restaurants, transport, personal services, etc.).

All this traffic of tertiary activities generates investment, employment and income that, at the local level, complements the primary agricultural activities, the traditional economic base, generating new horizons of activity. A different question is that this type of activity can offer the necessary productive base for population regeneration, which for the purposes of our study would be really interesting. It is still early to pronounce, but the data does not seem to indicate that the implementation of these activities per se is slowing down depopulation or enabling the arrival of new residents to the border municipalities to offset their negative vegetative growth. However, it is necessary to say that they can help (and a lot) to change mentality and to get out of that emotional situation of "resignation" and "lethargy" that seems to have dominated the social climate on the Hispanic side until recently. The territorial implementation of Tourism also seems to show the commitment of local and regional institutions to a territory abandoned to its fate until very recently, which is an indication that "something" is changing.

In any case, the few Spanish municipalities with the greatest resilience present a very specific profile: Strategic location, with good road and communication infrastructures that allow for better comparative accessibility, together with good provisions of public and private services for the population (educational health, security, legal) and facilities (schools, health and care centers, business parks, supermarkets, shops, banks, pharmacies, etc.). Along with all this, a diversified local economy coexists with a range of activities beyond traditional agriculture and job creation. In short, the nuclei with the greatest resilience are those with the highest quality of life, the most economically active and the referents in the territorial hierarchy of reference, which is very important with a view to possible future projections of the border territory and the design of policies. and countercyclical interventions against depopulation.

Although, as we have seen, depopulation is a common phenomenon on both sides of the border, producing in many cases this ‘recessive demographic continuity’, its impact seems to be much more intense on the Spanish side, where it reaches extreme values, as evidenced in Table 5. For this reason, to be exact, rather than depopulation, we should talk about desertification: a territory submerged in an advanced desertification process that needs economic and social institutional responses, to try to address the challenges that go beyond beyond the depopulation evidenced in our analysis.

**Table 5.** Summary Spanish Municipalities of Spanish-Portuguese North-Central border.

Densities h/km					
Province	Municipalities	-5h/km	-10h/km	+10h/km	+20
**Salamanca**	22	9	10	1	2
**Zamora**	15	7	5	3	0
**TOTAL**	**37**	**16**	**15**	**4**	**2**
Population losses compared to 1960 Census					
Province	Municipalities	+70%	+80%	+50	-50
**Salamanca**	22	8	8	4	2
**Zamora**	15	6	2	4	3
**TOTAL**	**37**	**14**	**10**	**8**	**5**
Percentage of population over 65 years of age as a total population					
Province	Municipalities	+40%	+50	-40	-25
**Salamanca**	22	11	5	4	1
**Zamora**	15	10	1	3	1
**TOTAL**	**37**	**21**	**6**	**7**	**2**

Source: Authors. Data from INE (2022)

In this particularly difficult context, since depopulation is a structural problem that conditions all aspects of life in these border territories, the implementation of cross-border administrative and institutional collaboration acquires fundamental importance, providing a multilevel system that requires the communitarization of Intergovernmental Relations (RIG) of each national territory, as well as of the public management of interdependent actions at the border within the framework of intergovernmental management (GIG) ( Agranoff, 1993; Elazar, 1987; Rose, 1984; Rose, 1985; Wright, 1981). In this way, it will be possible to respond to functional needs at community borders in a coordinated manner (both horizontally and vertically), effectively and legitimately.

### Cross-border institutional and administrative coordination: The case of the border between Castilla-León (Spain) and the Central and North regions of Portugal

***Cross border cooperation.*** We cannot omit the enormous relevance that cross border cooperation (CBC) has acquired today (About its definition, theoretical approach and role of individuals, see Table 6, Table 7, Table 8). As such a tool, aimed at overcoming border problems, the CBC has experienced great expansion in recent decades ( AEBR & EC, 1997), spreading both through the internal borders of the European Union ^11^ and through the external ones, reaching, based on the European experience, "those territories that had not spontaneously experienced this form of cooperation” ( Coletti, 2010: 160), even reaching the point of expanding the CBC to other continents ( Perkmann & Sum, 2002; Rhi-Sausi & Conato, 2009).

**Table 6.** Definitions of cross border cooperation.

	
"European Framework Agreement on Cross- border Cooperation between Communities or Territorial Authorities" Art 2 ( EOC-Tco, 1980)	"any concerted action aimed at reinforcing and developing neighborly relations between communities or territorial authorities ^10^ that belong to two or more contracting parties, as well as the celebration of agreements and appropriate arrangements for this purpose...".
Association of European Border Regions (AEBR and European Commission) (1997)	“direct neighborhood cooperation between regional and local institutions along a border, in all spheres of life and with the participation of all actors”
Perkmann (2003: 157)	"a collaboration between subnational authorities beyond national border limits"

Source: Podadera y Calderón (2018)

**Table 7.** Theoretical approach.

Approach	Theoretical Context	Vision of the Border
CROSS-BORDER APPROACH	Institutionalism	Borders as walls or barriers on one side and as bridges or contact zones on the other, offering opportunities for mutual understanding and integration. cross-border cooperation is seen as a common virtue.
FLOW APPROACH	Rational Choice Theories and Neoclassical Economics	Borders as obstacles to economic efficiency. By generating obstacles of physical distance, borders increase interaction costs, interrupting or hindering activity flows ( Nijkamp *et al.* (1990)
SOCIO-CULTURAL APPROACH (People Approach)	Social-Constructivismo	Borders (and border regions) are social constructions that are the result of processes of mental and symbolic creation carried out by different cross-border actors. The distinction between "us" and "them" or "others" is fundamental in the formation of the different border "identities", creating those "mental distances" that are constantly produced and reproduced ( Boman, 2005).

Source: Podadera y Calderón (2018)

**Table 8.** Roles of individuals in cross border cooperation (CBC).

	
CROSS-BORDER APPROACH	"homo contractis" ( Van Houtum, 2000: 64) individual or group that previously develops contacts that will lead them to conclude agreements and contracts aimed at satisfying their needs, later. Simultaneously, it is exploring new territories and opportunities in order to generate new contact and contract cycles.
FLOW APPROACH	“homo economicus”, profit maximizer and cost minimizer, so it will only have a favorable attitude towards cross- border cooperation if there is a gain or advantage in adopting said collective behavior ( Hernandez Ulate *et al.*, 2009).
SOCIO-CULTURAL APPROACH	"homo socialis" so that their group perceptions (positive or negative) about the CBC will be conditioned by the mutations that constantly take place in their changing "identity"

Source: Podadera y Calderón (2018)

From a theoretical-conceptual perspective, Van Houtum (2000), places cross-border cooperation within the so-called cross-border approach, a perspective that poses a dual or ambiguous vision of borders (either bridges or walls) for which positive management is essential. of them in the sense that they must be overcome: the borders “can be overcome and should be overcome” ( Van Houtum, 2000: 64), since the effects of overcoming the barrier effect will be very positive, particularly within of the territory of the European Union.

This cross-border ‘optimism’ is not shared by the so-called Socio-Cultural Approach ( Berg, 2000; Paasi, 1999; Wilson & Donnan, 1998) of border studies, an approach that does not consider borders as mere political lines of separation set by the state but rather “territorial markers” that delimit the physical space controlled by human groups of different identity and nationality ( Van Houtum, 2000:68), emphasizing the “national identity” and the emotional ties of each community with “its” territory. Identity that conditions human and social perceptions (not necessarily positive), of each community in relation to interactions with human groups beyond the border and, obviously, of cross-border cooperation.

Nor could the rise of the cross-border current of thought and its current dominant position, within border studies, be separated from the process of European integration that has given rise to the current EU. In these coordinates, cross-border cooperation has proven to be a basic instrument in the construction of that Europe "without borders" and cohesive, narrative, more or less rhetorical ( O'Dowd & Wilson, 1996), more or less romantic ( Von Malchus, 1975) that has fueled the process of European integration. Therefore, it is not surprising that in the EU coordinates the CBC is considered a kind of "common good", by eliminating both physical discontinuities and economic-fiscal, legal and cultural disparities between the two sides of the same border.

Hence, the emphasis that from the cross-border approach is given to the role played by the political-economic initiatives of the EU and, very specifically, to the support of programs such as INTERREG or the emergence of the various cross-border entities such as the Euroregions ( https://www.euroregio.eu/en/), a fundamental element of the so-called ‘Europe of the regions’ (another of the great narratives of the integration process) and to a greater or lesser extent referents to follow by cross-border initiatives ( Scott & Collins, 1997). Through cross-border cooperation embodied in the Euroregions, the border regions they go from being “passive spaces” on the European economic map to “active spaces” “key areas for crossborder policy development” ( Van Houtum, 2000: 64), integral parts of the resulting European economic transnational network ( Nijkamp, 1993). In the Euroregions, the institutionalization of the CBC has been achieved, which has allowed an effective and long-term projection of the initiatives promoted ( Coletti, 2010).

It should not be omitted from this ‘success’ story that the first experiences of cross-border cooperation in Europe arose in the mid-50s at the initiative of the municipal governments of the borders between the Netherlands and Germany, creating the so-called Euroregio Gronau-Emschede (1958), while at the initiative of economic and social agents, the cities of Basel (Switzerland), Mulhouse (France) and Friburg (Germany) led to the creation of the Regio Basilensis (1963). For this reason, institutionalized cross-border cooperation, as such "social innovation", arises in settled, peaceful, urbanized and populated border areas, that is, already "developed", where many elements of socioeconomic complementarity and cross-border continuity pre-existed, giving rise to a kind of cross-border regional awareness ( CE, 2000, Op. Cit.). This was not the case with other European borders, such as the Iberian, Hellenic, Italian-Slovenian, Balkanic or other Central-Eastern European borders ( Maya, 2007), asymmetric, rural, peripheral, unpopulated, heterogeneous or contested and insecure borders. Borders where that practical socio-economic homogeneity does not occur, nor that, to a greater or lesser extent, cultural continuity. Therefore, in principle, the preconditions did not exist to achieve smooth and successful cross-border cooperation.

### The Institutional organization of cross-border cooperation between the Autonomous Community of Castilla-Leon and the Central and Northern Portuguese Regions

Coinciding with the incorporation of Spain to the Council of Europe, the Treaty of Friendship and Cooperation between Spain and Portugal was signed in 1977, aimed at strengthening the ties of friendship and solidarity between the two countries, based on common interests. Simultaneously, ‘cross-border cooperation’ begins to be seen, referring to closer cooperation between territorial entities of Spain and Portugal, located in the border area, and which do not have competences in foreign relations.

In order to articulate the relations between these entities, within the Council of Europe the European Framework Convention for Cross-border Cooperation between Communities or Territorial Authorities was drafted in 1980, which was ratified by Spain in 1990. Following its stipulations, in 2004 the Treaty of Kingdom of Spain and the Portuguese Republic on cross-border cooperation between entities and territorial instances, commonly known as the Treaty of Valencia ^12^.

With the accession of Spain and Portugal to the European Union (1986) and the consequent disappearance of internal borders, relations between Spain and Portugal are intensifying from an institutional and socioeconomic point of view, contributing significantly to the emerging CBC that, little by little, is going to densify and deepen the Spanish-Portuguese relations at the regional, regional and local level. In this pro-cooperatio context with the Declarations, the Government of Castilla y León formally began its cooperation relations with the Northern Region of Portugal and the Central Region, respectively.

The formalization of the first manifestations of the CBC between the Autonomous Community of Castilla y León and the bordering Portuguese regions of the North and Center of Portugal began in 1990 with the signing of the Declarations of intent in this regard. In 1995, these relations were institutionalized by signing the Collaboration Protocols between the Spanish CCAA of Castilla y León with the Northern and Central regions of Portugal, respectively, giving rise to the constitution of two stable structures for the CBC: the Castilla y León Work Community. León-Centro de Portugal (1995) and the Community of Castilla y León-Norte de Portugal in the year 2000.

The Castilla y León-Centro de Portugal and Castilla y León-Norte de Portugal Work Communities are stable cross-border cooperation structures that link both territories and promote neighborly relations, recognizing the important historical, cultural, economic and geographical ties that unite them. With it, it is intended to contribute to the sustainable development of the territory of the ray and to favor the improvement of the living conditions of the citizens.

The entry into force of the Treaty of Valencia in 2004 will require the adaptation of the legal instruments that to date supported the cooperation relations between Castilla y León and the Portuguese regions of the Center and North of Portugal, signing the adaptation agreements with both regions in 2008 and 2009, respectively.

In 2009, a qualitative step was taken in the relations between Castilla y León and Portugal with the signing of the Memorandum of Understanding on Cross-border Cooperation between the Ministry of the Environment, Territory Planning and Regional Development of the Government of Portugal and the Junta de Castilla y León. It is the first of these characteristics to be signed between the Government of Portugal and a Spanish Autonomous Community.

### Organization and structure of work communities

***A) Castilla y León-Central Portugal Work Community (CENCYL).*** The Castilla y León-Centro de Portugal Work Community is a body without legal personality, which is organized into (Source): Presidency (including the Vice-Presidency), Plenary Council, Board of Directors, Secretariat and Sectoral Committees.

The Presidency is alternative, corresponding either to the Coordination and Regional Development Commission of the Portuguese Center (CCDRC), or to the Junta de Castilla y León, exercising the Vice Presidency that entity that does not hold the Presidency.

The Plenary Council is the plenary body of the Work Community, integrating the Presidency and the representatives of the Sectoral Committees. Meeting in plenary session, they approve the general action program of the Work Community and the biannual activity report.

The Board of Directors ensures the continuity of the activities of the Work Community. It integrates the Presidency and the General Coordinators. For its part, the Secretariat is ensured by the Cabinet of Cross-Border Initiatives (GIT) ^13^, a technical structure that has antennas in the headquarters of both regional governments.

Finally, the Sectoral and Territorial Committees bring together the political and administrative managers of the two regions, and representatives of other public and private organizations and entities, so that they can work in an organized manner on cross-border cooperation projects in the following thematic areas:

- Transport and Territorial Planning
- Natural, Cultural and Tourism Heritage
- Competitiveness and Innovation
- Promotion and Social Development
- [Territorial Committee] Salamanca-Beira Interior North

Regarding the cross-border cooperation activities carried out in this Work Community, it is worth noting, according to CENCYL ^14^:

➢ Cross-border cooperation micro-initiatives, which are those that have arisen in the eligible territory of both regions that, due to their financial dimension and the nature of their promoters, are not likely to submit an application for European territorial cooperation programs (language courses and other cultural events)
➢ Sectoral meetings. These are meetings of representatives of public administrations with competences in a specific matter, researchers, experts, agents and other representatives of civil society. Convened under the name of conferences, workshops, forums, their purpose is the coordination of initiatives, projects and action proposals for cooperation and exchange of experiences, as well as the increase in the number of cooperation projects, and the number of inhabitants of both regions benefited by these initiatives.
➢ Technical conferences, identification workshops, seminars or meetings on Cross-Border Context Costs or "those derived from the application of provisions established in regulations or other legal instruments, whose control corresponds exclusively to governmental or public entities or institutions and whose application includes the border territory between Spain and Portugal” (CENCYL 2020) ^15^.
➢ Publications and Exhibitions

The Castilla y León-Central Portugal Work Community has a catalog of publications on cross-border issues. It also has photographic collections that it gives out free of charge to entities that request it to be exhibited in towns in Castilla y León, North and Central Portugal, in order to disseminate landscape, ethnographic or other aspects of La Raya.

***B) Castilla y León-North Portugal Work Community (NORCYL).*** The Castilla y León-North Portugal Work Community (NORCYL) is a body without legal personality, formally constituted in the year 2000 by both regions. Its structure and organization are quite similar to CENCYL.

The Work Community is structured in: Presidency (including the Vice Presidency), Plenary Council, Board of Directors, Secretariat and Sectoral Committees. The Presidency is, alternatively, falling either to the Commission for Coordination and Regional Development of the North (CCDRN), or to the Junta de Castilla y León, exercising the Vice Presidency that entity that does not hold the Presidency.

The Plenary Council is the plenary body of the Work Community, integrating the Presidency and the representatives of the Sectoral Committees. Meeting in plenary session, they approve the general action program of the Work Community and the biannual activity report.

The Board of Directors ensures the continuity of the activities of the Work Community. It integrates the Presidency and the General Coordinators. For its part, the Secretariat is ensured by the Cabinet of Cross-Border Initiatives (GIT) of Castilla y León, and the Divisão de Coordenação de Projetos e Redes Institucionalis del Norte de Portugal, whose mission is to support the functioning of the Work Community.

The Sectoral and Territorial Committees bring together the political and administrative managers of the two regions, and representatives of other agencies and public and private entities, so that they can work in an organized manner on cross-border cooperation projects in the following thematic areas:

➢ Competitiveness, Innovation, Employment and Training
➢ Natural Heritage, Cultural Heritage and Tourism
➢ Accessibility, Logistics and Territorial Planning
➢ Institutional cooperation and social policies
➢ Structuring project "Duero-Douro"
➢ [Territorial Committee] Braganza-Zamora, Salamanca-Douro Superior, Duero-Douro, Zasnet

The cross-border cooperation activities of this Work Community (NORCYL) ^16^ are very similar to those of CENCYL (Micro-initiatives, Meetings, Context Costs, Publications and Exhibitions).

Both Work Communities have promoted the Fronteira Project for the institutional strengthening of the work communities themselves. Finally, both Communities, Norcyl and Cencyl, have expressed their willingness to join REDCOT ^17^, along with some thirty entities with a similar vocation: Work Communities, European Groupings for Territorial Cooperation (EGTC), cross-border EURES, Eurocities, among others.

## Discussion

As we have been able to verify, the CBC of the Central and Northern Regions of Portugal with the Autonomous Community of Castilla y León appears clearly oriented towards the connection between local administrations, at the regional and municipal level, to their daily joint work, to the forum, to the debate, to the cultural approximation (in all its senses, linguistic, literary, gastronomic, landscape, anthropological, etc.) to the other side of the Border, to the knowledge of the environment and of the resources of the territory, the culture and identity of the ‘other’. In short, a CBC basically oriented towards contact (and meeting) between administrations and border populations (regional, provincial and municipal) and to the revitalization of the border context, but very little oriented towards solving specific problems; and if we put it in relation to a structural, difficult and complex problem such as depopulation, the CBC is simply overwhelmed.

It is interesting and significant the effort of territorial diffusion that with respect to the theme of depopulation has been carried out from the local instances (local administrations, territorial associations, etc.) in the cross-border area. In this sense, numerous initiatives and proposals come from the Castilian-Leonese-central-northern cross-border area, from the Vilar Formoso-Fuentes de Oñoro Eurocity ( García, 2019), to the 'Memorandum of understanding to adopt an Iberian strategy against depopulation and aging in the border area ( Martínez, 2020) resulting from the 2018 Hispano-Lusa summit, or the "Declaration of Urgency of the Celtic Strip" ( Zamora News, 2021), promoted by cross-border associations such as Viriatos and La Raya- To Raia, claiming, given the magnitude of the demographic problem, an Integrated Territorial Investment ^18^, so that all European funds can be brought together, promoting participatory local development and the prominence of local action groups. For the execution of this Integrated Territorial Investment, each State should organize a multilevel governance system based on four pillars: public, regional and local authorities; economic and social partners; civil society organizations and research organizations and universities.

For its part, considering the limits of the study carried out, It is never easy to compare Spain and Portugal, even though there are so many similarities (EU members, shared past, joint future, etc.) given that both their administrative structures and their territorial organization are substantially different: While Spain today is a country Strongly decentralized, Portugal maintains high levels of centralization, with the ‘Regiões’ having an essentially statistical functionality (for EU statistical purposes).

For this reason, in order to carry out the Study effectively, we have taken advantage of the synergies derived from the Nomenclature of Territorial Units for Statistics (NUTS), which allows us to harmonize the territory under study, given that the Portuguese NUTSIII would be equivalent to the Spanish provinces (see Table 1)

In this particularly difficult context, since depopulation is a structural problem that conditions all aspects of life in these border territories, the implementation of cross-border administrative and institutional collaboration acquires fundamental importance, providing a multilevel system ( Faludi, 2012) that requires the communitarization of Intergovernmental Relations (RIG) ( Agranoff, 2010; Phillimore, 2013) of each national territory, as well as of the public management of interdependent actions at the border within the framework of intergovernmental management (GIG). In this way, it will be possible to respond to functional needs at community borders in a coordinated manner (both horizontally and vertically), effectively and legitimately.

## Conclusions

The analysis of the empirical evidence of institutional coordination in the cross-border area of the Castilian-Leonese borders with the Central and North regions of Portugal, shows a preferably relational and contact orientation, but little oriented to the resolution of the structural problems that affect to the cross-border territory, as the case of depopulation.

In this sense, cross-border cooperation between both sides of the border has been generating a network of joint institutional interactions (Working Communities, Initiative Groups, Joint Municipal Associations...) between the territorial administrations, aimed fundamentally at mutual understanding and facilitation of daily cross-border contacts, where inter-governance is still very incipient, lacking the operational dimension so necessary in modern management schemes in territories as complicated as borders.

The results of the study carried out show a very complicated demographic situation in terms of the loss of population with respect to the base year of 1960, wich tends to exceed, in many cases, 60% of the entire population, evidenced in the low population densities observed along the entire border line and also observing in high levels of aging.

Likewise, the study evidences the existence of a recessive continuum on both sides of the border with sparsely populated territories with a scarce and aged population critical mass, a situation derived both from both: the serious population losses observed since 1960, and the net predominance of deaths over births. All of the above is mainly related to sectoral economic specialization and, therefore, to the economic progression of the territory.

The existence of this recessive demographic continuum, which extends to both sides of the border, causes the need to face the depopulating phenomenon from a transnational (and European) perspective, in the sense that ‘national’ solutions to borderland depopulation will not be able to offer consistent answers to a phenomenon as complex as depopulation. For this, it is necessary to reinforce the existing cross-border cooperation, providing it with greater operability at the level of institutional and administrative coordination, deepening the multilevel governance of the territory.

So far, only territorial dissemination and awareness actions have been observed regarding the issue of depopulation, carried out from local authorities (City Halls, Chambers, business associations...). But such suggestive initiatives must have operational continuity: knowledge of the problems that affect the territory is very important, but it is equally important to offer concrete solutions.

## Data availability

The data used in this study was obtained from public databases including the National Institute of Statistics (INE) and the Statistics Portugal database of the Portuguese National Institute of Statistics (INE.pt) as outlined in the methods section.

The References section specifies the source of information for these data based on the publication of Curvelo (2020): https://www.jornaldenegocios.pt/economia and within this go to: https://www.jornaldenegocios.pt/economia/detalhe/em-quase-metade-dos-concelhos-25-da-populacao-e-idosa-veja-no-mapa-a-situacao-no-seu

The institutions involved are INE.pt and ISEG - Lisbon School of Economics & Management
