# Supplementary material for: Borders, cross-border cooperation and depopulation: the case of the Spanish-Portuguese North-Central Border
Source: Open Res Eur. 2023 Nov 20;3:200. [Version 1] doi: 10.12688/openreseurope.16319.1 (PMC11066538; doi:10.12688/openreseurope.16319.1)
Supplement: Peer review history

# Reviewer report: Reviewer response for version 1

**DOI:** 10.21956/openreseurope.17616.r39653

**Published:** 2024-05-07

**Authors:** Giovanni Ruggieri

**Affiliations:** 1 Universita degli Studi di Palermo, Palermo, Sicily, Italy

The scientific article deals with the issue of cross-border cooperation to address the dynamics of depopulation in the central-northern Spanish-Portuguese border.

 The abstract provides a concise overview of the article, effectively underlining the need for joint efforts between national and local actors to improve the quality of life of border populations. 

 The introductory paragraph effectively defines the context of the study and clarifies the rationale for examining the issue within the institutional cooperation framework. 

 Reference is made to the study methods for the period 1960- 2020. The use of three variables essential for assessing population evolution, critical population mass and ageing, is appropriate to achieve the research objectives.

 In the results section, the article effectively presents the study's results, highlighting the existence of a recessive demographic continuum. Then, in the discussion of the implications of these results for addressing depopulation from a transnational and European perspective, depth is added to the analysis. 

 The conclusions underline the need to strengthen cross-border cooperation and the demand for greater operational institutional and administrative capacity.

 The research is well conducted, and the results contribute to the existing regional development and governance literature. However, minor reviews are recommended to improve the study's clarity, transparency and practical implications.

If applicable, is the statistical analysis and its interpretation appropriate?

Yes

Is the study design appropriate and is the work technically sound?

Yes

Is the work clearly and accurately presented and does it engage with the current literature?

Yes

Are the conclusions drawn adequately supported by the results?

Yes

Are sufficient details of methods and analysis provided to allow replication by others?

Yes

Are all the source data and materials underlying the results available?

Yes

Reviewer Expertise:

Giovanni Ruggieri is an associate professor of Applied Economics and holds the chair of Economics of Tourism and Tourism Industries at the University of Palermo. Founder and editor of the International Journal of Island Research at Dublin Technology University, he is the author of several scientific articles on the analysis of the economic impact,  social networks, local development, cultural and island tourism and has drawn up several management plans for UNESCO.  He holds the position of President of the Observatory of Tourism of the European Islands (OTIE) and is a member of the International Association of Scientific Experts of Tourism (AIEST), the International Association of Economists of Tourism (IATE), the Italian Society of Economics (SIE). giovanni.ruggieri@unipa.it

I confirm that I have read this submission and believe that I have an appropriate level of expertise to confirm that it is of an acceptable scientific standard.

# Reviewer report: Reviewer response for version 1

**DOI:** 10.21956/openreseurope.17616.r36340

**Published:** 2024-05-02

**Authors:** Giovanni Ruggieri

**Affiliations:** 1 Universita degli Studi di Palermo, Palermo, Sicily, Italy

This insightful article demonstrates a keen understanding of the pivotal role that cross-border cooperation plays in addressing the multifaceted challenges of depopulation. The emphasis on a transnational and European perspective is commendable, recognizing the limitations of purely 'national' solutions in tackling depopulation's intricate and pervasive nature. The meticulous descriptive analysis conducted on the Spanish-Portuguese north-central borderline, spanning the period from 1960 to 2020, provides a robust foundation for the conclusions drawn.

 The call for reinforced cross-border cooperation and enhanced operability at the institutional and administrative levels underscores the importance of coordinated efforts. The focus on deepening multilevel governance reflects a nuanced approach to address depopulation, acknowledging the need for comprehensive strategies involving governments national and local actors on both sides of the border.

 This article contributes valuable insights to the ongoing discourse on depopulation and provides a constructive pathway forward by recommending economic policy interventions within the framework of cross-border institutional cooperation. The commitment to improving the quality of life for populations in border areas is evident throughout, making this study a significant and timely contribution to the field.

If applicable, is the statistical analysis and its interpretation appropriate?

Yes

Is the study design appropriate and is the work technically sound?

Yes

Is the work clearly and accurately presented and does it engage with the current literature?

Yes

Are the conclusions drawn adequately supported by the results?

Yes

Are sufficient details of methods and analysis provided to allow replication by others?

Yes

Are all the source data and materials underlying the results available?

Yes

Reviewer Expertise:

NA

I confirm that I have read this submission and believe that I have an appropriate level of expertise to confirm that it is of an acceptable scientific standard.

# Reviewer report: Reviewer response for version 1

**DOI:** 10.21956/openreseurope.17616.r36341

**Published:** 2023-12-15

**Authors:** Mario Kölling

**Affiliations:** 1 Fundación Manuel Giménez Abad, Zaragoza, Spain

The aim of the text is to analyse the extent to which cross-border practices have influenced border demographic dynamics. The authors aim to make a new contribution to the academic debate that will enrich critical reflection and debate on the issue of depopulation, with a view to proposing recommendations for economic policy, within a framework of cross-border institutional cooperation and inter- and intra-governmental coordination.

 This is a very interesting topic. I would like to briefly give a few hints that could help to improve the text and make it more understandable for the readers.

 Since the authors consider their text as a research article, I would recommend structuring the text in this sense, starting with a conceptual framework, a literature review, a research question and a hypothesis.

 Overall, the article is structured in two parts:

- The first part is a descriptive analysis of the evolution of population indicators over the period 1960-2020. The data is well presented, but I miss a literature review on the topic. Furthermore It should be clearer why the authors choose these indicators and this time frame. The authors should include a rational on the territorial unit of analysis: NUTS 3; two large sections border territories, or NUTS II; the authors should explain in more detail why they chose these different territorial units, This is particularly important for a clear comparison.
- Regarding the results of the first part, the collapse of the population in Spanish border municipalities in the period 1960-2020 is very evident, but has been highlighted by several studies, the question would be whether there are specific characteristics of the border regions? If so, the authors should compare this finding with the situation in other Spanish regions. The same would be useful for Portugal. It would make sense to establish two categories "border regions" and "non-border regions". If border regions are more affected by depopulation than "non-border regions", it would give a strong motivation to analyse cross-border cooperation.
- The authors claim that the Portuguese part of the Raya Centro-Norte is resisting the depopulation pressure better than the Spanish part, they should present some more evidence of this.
- The authors argue that there is a Portuguese strategy for urban or pseudo-urban border areas, either to "anchor" the population, but there is no reference to this strategy. It should be clarified whether this national strategy is only for one local entity, or why it does not work for the rest of the units. There may also have been several strategies on the Spanish side, why did they not work, why were former INTERREG programmes not successful?
- It should be clarified why the INE data have been supplemented with the database of institutes specialised in territorial statistics. The same for the Portuguese National Statistical Institute.
- In summary, the authors could elaborate on the specific characteristics of border regions and their research methodology.
- The second part starts with a theoretical framework, this should be at the beginning and linked to the study on depopulation. The second part should be more concentrated on the question of what CBC means for the problem of depopulation. Especially regarding the Castilla y León-Central Portugal Work Community and Castilla y León-North Portugal Work Community (NORCYL). The authors should increase the empirical evidence of the CBS in relation to cross border regions, .e.g. evolution of the budget …
- The authors mentioned the concepts of multi-level system, Europe of regions and Euroregions, there should be a better definition of these concepts, it may become clear that there are important conceptual differences between these terms.
- The first analysis is based on nuts 3 the second part concentrated on nuts 2, the discussion should include a few words on the implications of this fact for the comparison.
- In general terms both parts - a descriptive analysis of the population situation in the municipalities of the North-Central Spanish-Portuguese border and the descriptive analysis of institutional coordination in cross-border areas - should be better connected.
- Above all, the interrelationship between population and CBC should be made clearer. The author could also include some economic policy recommendations, as mentioned in the introduction.
- The text would benefit from a linguistic revision, some expression should be more precise:
- “It is never easy to compare Spain and Portugal, even though there are so many similarities (EU members, shared past, joint future, etc.)”
- “In short, the necessary cocktail for a ‘better’ life that, on the one hand, ‘retains’ the current residents, preventing them from abandoning the territory and, on the other, ‘attracts’ the older population to these ‘urban functionality’ areas. young and ready.”
- “This bet of the Portuguese State for the border urban or pseudo-urban areas […].“

If applicable, is the statistical analysis and its interpretation appropriate?

Yes

Is the study design appropriate and is the work technically sound?

Partly

Is the work clearly and accurately presented and does it engage with the current literature?

Partly

Are the conclusions drawn adequately supported by the results?

Partly

Are sufficient details of methods and analysis provided to allow replication by others?

Yes

Are all the source data and materials underlying the results available?

Yes

Reviewer Expertise:

federalism, multi-level governance, EU budget and redistributive policies

I confirm that I have read this submission and believe that I have an appropriate level of expertise to confirm that it is of an acceptable scientific standard, however I have significant reservations, as outlined above.

# Reviewer report: Reviewer response for version 1

**DOI:** 10.21956/openreseurope.17616.r36342

**Published:** 2023-12-14

**Authors:** Roberto Cippitani

**Affiliations:** 1 Universita degli Studi di Perugia, Perugia, Umbria, Italy

- The  work is clearly and accurately presented, with the conclusions consistent with the hypothesis and based on data. Yes, both the conclusions and the hypotheses are consistent with a basic correlation existing between them. Likewise, the paper's database comes from official statistical sources of both countries INE.es and INE.pt.  

 - The study design is appropriate and the work is technically sound. The study tries to explain the depopulation phenomenon in the Spanish-Portuguese border framework. in this sense, the whole study is designed according to a two sides border scheme (tables,  data, general information, etc.) trying to explain the border area as a single territory.     

 - The paper clearly shows data and information necessary for analysis carried out by others. Yes, broadly speaking the paper offers a useful amount of information (economic-demographic and geographic at different levels: local municipalities, provinces, departments, regions) which could be used for other researchers as a primary source.

 - The article makes reference to the sources of data used and which may be easily reached in order to reuse them. Yes, in the paper's framework normally the statistical sources are correctly established, being official and institutional sources is relatively easy to get them.   

 - The statistical analysis and its interpretation is appropriate.

 Yes, usually authors use descriptive statistics that greatly facilitate the reading and understanding of the analysis and its preparation for a social science audience to whom the article is directed

If applicable, is the statistical analysis and its interpretation appropriate?

I cannot comment. A qualified statistician is required.

Is the study design appropriate and is the work technically sound?

Yes

Is the work clearly and accurately presented and does it engage with the current literature?

Yes

Are the conclusions drawn adequately supported by the results?

Yes

Are sufficient details of methods and analysis provided to allow replication by others?

Yes

Are all the source data and materials underlying the results available?

Yes

Reviewer Expertise:

Law of economics

I confirm that I have read this submission and believe that I have an appropriate level of expertise to confirm that it is of an acceptable scientific standard.
